# Supplementary material for: Multi‐dataset identification of innovative feature genes and molecular mechanisms in keratoconus
Source: J Cell Mol Med. 2024 Sep 19;28(18):e70079. doi: 10.1111/jcmm.70079 (PMC11412914; doi:10.1111/jcmm.70079)
Supplement: Supplementary file 2 — Material S2. [file JCMM-28-e70079-s002.zip › 7.GSEA analysis/6.SNORA74B/KEGG_PYRUVATE_METABOLISM.html]

Details for gene set KEGG\_PYRUVATE\_METABOLISM[GSEA]

|  || Dataset | SNORA74B.group.cls#h\_versus\_l |
| Phenotype | group.cls#h\_versus\_l |
| Upregulated in class | l |
| GeneSet | KEGG\_PYRUVATE\_METABOLISM |
| Enrichment Score (ES) | -0.36482447 |
| Normalized Enrichment Score (NES) | -1.4128484 |
| Nominal p-value | 0.11787819 |
| FDR q-value | 0.3216061 |
| FWER p-Value | 0.887 |
Table: GSEA Results Summary

  

Fig 1: Enrichment plot: KEGG\_PYRUVATE\_METABOLISM      
 Profile of the Running ES Score & Positions of GeneSet Members on the Rank Ordered List

  

| SYMBOL | TITLE | RANK IN GENE LIST | RANK METRIC SCORE | RUNNING ES | CORE ENRICHMENT || 1 | LDHAL6A | na | 4598 | 0.239 | -0.0675 | No |
| 2 | ME3 | na | 5679 | 0.202 | -0.0434 | No |
| 3 | HAGHL | na | 9706 | 0.099 | -0.1326 | No |
| 4 | ACSS1 | na | 10420 | 0.085 | -0.1301 | No |
| 5 | ACACA | na | 11245 | 0.071 | -0.1345 | No |
| 6 | ACOT12 | na | 11586 | 0.066 | -0.1265 | No |
| 7 | DLD | na | 12649 | 0.051 | -0.1431 | No |
| 8 | ALDH3A2 | na | 13104 | 0.046 | -0.1437 | No |
| 9 | PDHA2 | na | 13714 | 0.039 | -0.1506 | No |
| 10 | LDHD | na | 14845 | 0.026 | -0.1761 | No |
| 11 | PC | na | 15064 | 0.024 | -0.1759 | No |
| 12 | ACACB | na | 15478 | 0.020 | -0.1824 | No |
| 13 | ACAT2 | na | 15497 | 0.020 | -0.1776 | No |
| 14 | GRHPR | na | 16188 | 0.013 | -0.1940 | No |
| 15 | ACAT1 | na | 16541 | 0.009 | -0.2017 | No |
| 16 | PDHB | na | 16643 | 0.008 | -0.2025 | No |
| 17 | MDH2 | na | 16764 | 0.007 | -0.2041 | No |
| 18 | ACSS2 | na | 16817 | 0.006 | -0.2039 | No |
| 19 | ACYP2 | na | 17263 | 0.002 | -0.2161 | No |
| 20 | ME2 | na | 17869 | -0.004 | -0.2324 | No |
| 21 | PDHA1 | na | 18249 | -0.009 | -0.2409 | No |
| 22 | HAGH | na | 19679 | -0.025 | -0.2753 | No |
| 23 | MDH1 | na | 19809 | -0.026 | -0.2718 | No |
| 24 | ALDH7A1 | na | 19848 | -0.027 | -0.2656 | No |
| 25 | LDHC | na | 20197 | -0.030 | -0.2673 | No |
| 26 | GLO1 | na | 20350 | -0.032 | -0.2630 | No |
| 27 | LDHB | na | 21112 | -0.041 | -0.2736 | No |
| 28 | PKM | na | 24276 | -0.085 | -0.3416 | Yes |
| 29 | LDHA | na | 24376 | -0.087 | -0.3207 | Yes |
| 30 | DLAT | na | 24417 | -0.088 | -0.2980 | Yes |
| 31 | PKLR | na | 24696 | -0.093 | -0.2807 | Yes |
| 32 | PCK1 | na | 25015 | -0.099 | -0.2629 | Yes |
| 33 | ALDH9A1 | na | 25479 | -0.107 | -0.2471 | Yes |
| 34 | ME1 | na | 26640 | -0.131 | -0.2449 | Yes |
| 35 | LDHAL6B | na | 26958 | -0.139 | -0.2162 | Yes |
| 36 | ALDH1B1 | na | 27039 | -0.140 | -0.1803 | Yes |
| 37 | ACYP1 | na | 28355 | -0.175 | -0.1705 | Yes |
| 38 | AKR1B1 | na | 31422 | -0.290 | -0.1800 | Yes |
| 39 | PCK2 | na | 32780 | -0.374 | -0.1171 | Yes |
| 40 | ALDH2 | na | 34359 | -0.636 | 0.0106 | Yes |
Table: GSEA details [plain text format]

  

Fig 2: KEGG\_PYRUVATE\_METABOLISM      
 Blue-Pink O' Gram in the Space of the Analyzed GeneSet

  

Fig 3: KEGG\_PYRUVATE\_METABOLISM: Random ES distribution      
 Gene set null distribution of ES for **KEGG\_PYRUVATE\_METABOLISM**
